# Molecular Mechanism Underlying the Plant NRT1.1 Dual-Affinity Nitrate Transporter

**DOI:** 10.3389/fphys.2015.00386

**Published:** 2015-12-18

**Authors:** Ji Sun, Ning Zheng

**Affiliations:** ^1^Department of Pharmacology, University of WashingtonSeattle, WA, USA; ^2^Howard Hughes Medical Institute, University of WashingtonSeattle, WA, USA

**Keywords:** NRT1.1, dimer, dual-affinity, nitrate transporter, major facilitator superfamily, transceptor

## Abstract

Nitrate (${\text{NO}}_{3}^{-}$) is one of the most important sources of mineral nitrogen, which also serves as a key signaling molecule for plant growth and development. To cope with nitrate fluctuation in soil that varies by up to four orders of magnitude, plants have evolved high- and low-affinity nitrate transporter systems, consisting of distinct families of transporters. Interestingly, the first cloned nitrate transporter in *Arabidopsis*, NRT1.1 functions as a dual-affinity transporter, which can change its affinity for nitrate in response to substrate availability. Phosphorylation of a threonine residue, Thr101, switches NRT1.1 from low- to high-affinity state. Recent structural studies have unveiled that the unmodified NRT1.1 transporter works as homodimers with Thr101 located in close proximity to the dimer interface. Modification on the Thr101 residue is shown to not only decouple the dimer configuration, but also increase structural flexibility, thereby, altering the substrate affinity of NRT1.1. The structure of NRT1.1 helps establish a novel paradigm in which protein oligomerzation and posttranslational modification can synergistically expand the functional capacity of the major facilitator superfamily (MFS) transporters.

## Introduction

Nitrate (${\text{NO}}_{3}^{-}$) is critical for plants, both as a primary nutrient and as an important signaling molecule (Crawford, 1995; Krouk et al., 2010a). Nitrogen (N) is a key constituent of nucleotides and amino acids, thus essential for life. In plants, about 2–5% of the dry biomass is made up of nitrogen, which is largely acquired by plant roots in the form of nitrate (Xu et al., 2012). Nitrate also functions as a critical signaling ion and regulates many aspects of plant growth and development (Castaings et al., 2011; Bouguyon et al., 2012), including nitrate-related gene expression (Wang et al., 2003), root architecture (Forde, 2014), seed dormancy (Alboresi et al., 2005), and flowering time (Castro Marín et al., 2011).

Active nitrate uptake through membrane transporters by plant roots represents the key first step of nitrogen acquisition (Dechorgnat et al., 2011). As sessile organisms, plants have evolved sophisticated nitrate transporter systems in response to the fluctuating nitrate environments (Wang et al., 2012). In *Arabidopsis thaliana*, one of the most well studied nitrate transporters is NRT1.1 (CHL1 or NPF6.3), which is a multifunctional protein with a crucial role in both nitrate acquisition and signaling. Firstly, NRT1.1 is a dual-affinity transporter, which can facilitate nitrate assimilation over a wide range of nitrate concentrations (Liu et al., 1999). Secondly, NRT1.1 has recently been shown to serve as a nitrate sensor, regulating the gene expression of other nitrate transporters such as NRT2.1 (Krouk et al., 2006; Ho et al., 2009). Last by not least, NRT1.1 also contributes to the nitrate-regulated auxin translocation besides nitrate transport and sensing (Krouk et al., 2010b). In this review, we will mainly focus on the transporter functions of NRT1.1 and summarize recent structure-function studies, which have shed light on the molecular mechanism underlying its dual-affinity activity.

### Nitrate transporters in *Arabidopsis*

To cope with the fluctuation of nitrate level, plants have evolved two complementary nitrate transporter systems with distinct kinetics properties (Forde, 2000; Nacry et al., 2013; Krapp et al., 2014). The low-affinity transporter system (LATS), which consists members of the NRT1/PTR family (recently renamed as NPF family) (Steiner et al., 1995; Léran et al., 2014), drives nitrate uptake at millimolar concentration. This family shares sequence homology to SLC15/PTR/PepT/POT family of peptide transporter family in animal. In plants, the NRT1/PTR family has functionally diverged with individual members recognizing different substrates including peptides (Tsay et al., 2007), plant hormones (Krouk et al., 2010b), glucosinolates (Nour-Eldin et al., 2012), and nitrate (Tsay et al., 1993). The high-affinity transporter system (HATS), comprising the NRT2/NNP family, facilitates nitrate uptake with Michaelis constant (K_*m*_) value in the micromolar range. Studies suggest that some NRT2 members require a second gene product for their functional activity. In the case of NRT2.1, NAR2 is needed to mediate its nitrate uptake (Kotur et al., 2012).

NRT1/PTR and NRT2/NNP together constitute a large NRT transporter family in plants, which are proton-coupled symporters and belong to the major facilitator superfamily (MFS) (Pao et al., 1998). Together there are 53 NRT1 genes and 7 NRT2 genes in *Arabidopsis* (Tsay et al., 2007). Besides ensuring the root capacity of nitrate uptake, NRT transporters are also involved in subsequent loading and unloading of nitrate to and from the xylem vessels, allowing its distribution to aerial organs or its remobilization from old leaves (Chiu et al., 2004; Lin et al., 2008; Fan et al., 2009; Wang and Tsay, 2011).

### NRT1.1 is a dual-affinity transporter

The *Arabidopsis* NRT1.1 (CHL1 or NPF6.3) protein is the founding member of the NRT1/PTR family. It was firstly cloned as a gene product that is responsible for chlorate sensitivity in *Arabidopsis* (Tsay et al., 1993). The NRT1.1 protein contains 12 membrane-spanning segments and confers proton-coupled nitrate transport activity.

Interestingly, NRT1.1 is essential for both high- and low-affinity nitrate absorptions in *Arabidopsis*. It presents a biphasic uptake curve in response to environmental nitrate availability, and thus functions as a dual-affinity transporter (Huang et al., 1996; Wang et al., 1998; Liu et al., 1999; Liu and Tsay, 2003). NRT1.1 shares sequence homology with members of the NRT1/PTR family that constitutes the LATS, and was initially shown to be a low-affinity nitrate transporter. Later, it was found that plants with *nrt1.1* mutation were also defective in high-affinity nitrate uptake, suggesting that NRT1.1 might be a dual-affinity transporter. Further experiments in plants and oocytes demonstrate that the uptake curve of NRT1.1 is biphasic with a Km of ~50 μM for high-affinity phase of uptake and ~4 mM for the low-affinity phase (Liu and Tsay, 2003).

The mode of action of the dual-affinity function of NRT1.1 is regulated through phosphorylation modification on a key threonine residue, Thr101 (Liu and Tsay, 2003). Thr101 is located at the intracellular side between the third and fourth transmembrane helix (TM) of NRT1.1. When phosphorylated by the CIPK23 kinase (Ho et al., 2009), NRT1.1 functions as a high-affinity nitrate transporter. When unphosphorylated, it works as a low-affinity transporter. Furthermore, mutations of Thr101 preventing or mimicking phosphorylation can effectively convert the dual-affinity transporter into a monophasic low-affinity or high-affinity transporter, respectively. This regulatory mechanism of NRT1.1 allows for the rapid adaption to changing nitrate levels.

### Molecular basis of the dual-affinity function

**Figure 1:**
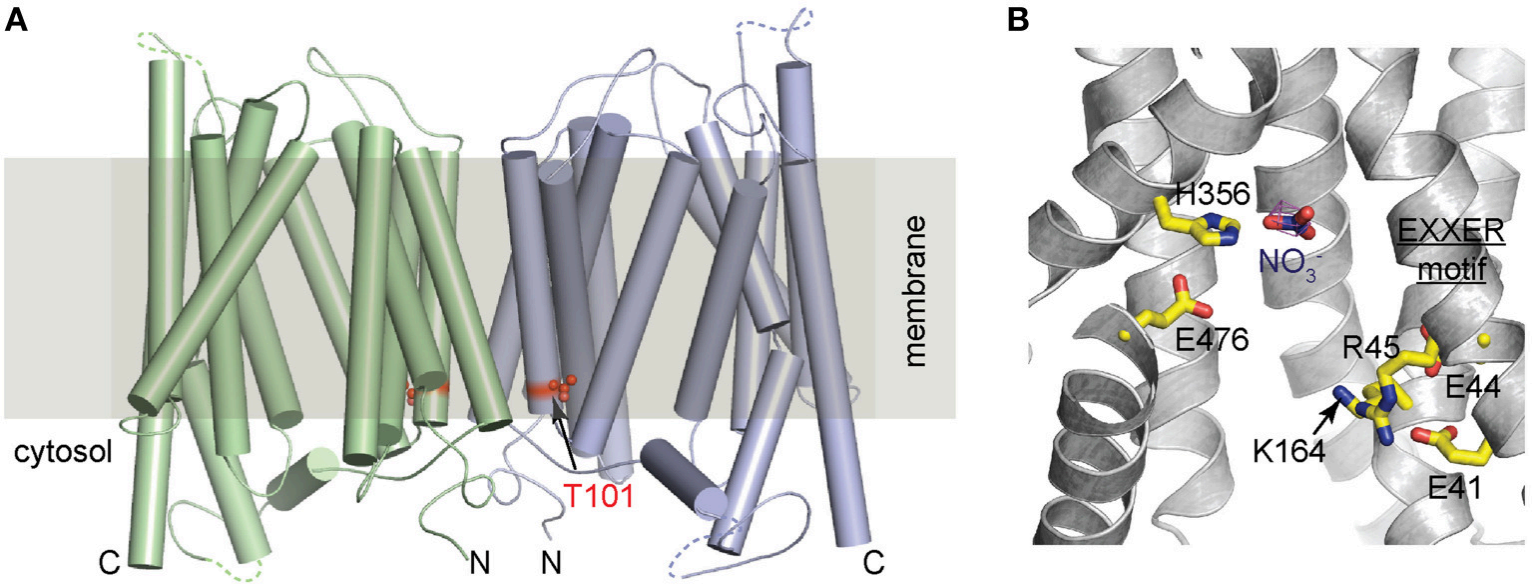
**Crystal structure of NRT1.1**. **(A)** A cylinder representation of the NRT1.1 dimer with Thr101 shown as red spheres. The two monomers are colored in pale green and light blue. Within each monomer, the amino- and carboxyl-terminus are indicated by N and C. **(B)** Key residues involved in substrate binding and proton coupling. Nitrate and the side chain of the neighboring histidine, His356, EXXER motif, Glu476, and Lys164 are shown as sticks.

How does Thr101 phosphorylation switch the transporter affinity of NRT1.1? Recently, two independent studies revealed the crystal structures of unmodified NRT1.1, shedding light on the molecular basis of the working mechanism of this transporter (Parker and Newstead, 2014; Sun et al., 2014). In both studies, NRT1.1 was crystallized with two molecules in each asymmetric unit (ASU), despite the different polypeptide boundaries, crystallization conditions, and space groups (Figure 1A). The two molecules in the ASU are almost identical to each other and adopt the canonical MFS fold, which is characterized by 12 transmembrane helices (TMs) with a central linker connecting the amino-terminal (TM1-TM6) and carboxyl-terminal (TM7-TM12) helix bundles (Law et al., 2008; Yan, 2013). The transporter is captured in the inward conformation with the substrate-binding site accessible from the cytosol side.

Both *in vitro* biochemical studies and cell-base fluorescence resonance energy transfer (FRET) assays suggest that the NRT1.1 dimer could be physiologically relevant (Sun et al., 2014). First of all, NRT1.1 dimer is observed in two different crystal forms. The two adjacent non-crystallographic related NRT1.1 molecules are in the same orientation with their amino-terminal halves facing and interacting with each other. This overall topology is perfectly compatible with its transporter function in the cell membrane (Figure 1A). Secondly, the dimer interface, burying a surface area of more than 2160 Å^2^, has high shape-complementarity that is considered as an important determinant of helix packing specificity in membrane or micelles (Fleming et al., 1997; Mackenzie and Fleming, 2008; Chen et al., 2009). Thirdly, *in vitro* crosslinking experiments show that transient dimer of NRT1.1 could form in detergent solutions. Consistent with these *in vitro* observations, cell-based FRET assay also demonstrates that NRT1.1 oligomerizes in the membrane of *Xenopus* oocytes, where the dual-affinity function of NRT1.1 can be recapitulated.

More importantly, the oligomerization state of NRT1.1 could be affected by the phosphorylation modification on NRT1.1 (Sun et al., 2014). In the crystal structure, the key residue, Thr101 is buried in a hydrophobic pocket that is located right next to the dimer interface (Figure 1A). Phosphorylation of Thr101 was predicted to introduce electrostatic and conformational changes in its vicinity and disrupt the dimeric configuration of NRT1.1. This prediction was confirmed by oocyte-base FRET assays. Both wild-type NRT1.1 and the phosphorylation defective mutant NRT1.1-T101A generate robust and comparable FRET signals. In contrast, the phosphomimetic mutation, NRT1.1-T101D failed to show any significant signal, indicating a spatial separation of the two monomers. These data suggest a phosphorylation-dependent dimerization switching mechanism for the dual-affinity transporter: unmodified NRT1.1 forms structurally coupled dimers and works as a low-affinity transporter, whereas phosphorylated NRT1.1 undergoes dimer decoupling and adopts a high-affinity state.

Besides decoupling the dimer, phosphorylation on Thr101 also alters the transporter properties of each monomer (Parker and Newstead, 2014). NRT1.1-T101D has a lower melting temperature, indicating increased structure flexibility. Meanwhile, the transport rate of the phosphomimetic variant is also higher than wild-type protein by 2.8-folds in the liposome-base uptake assay. Therefore, enhanced structure flexibility of NRT1.1 through Thr101 phosphorylation leads to an increased transporter rate, which results in a lower K_m_.

**Figure 2:**
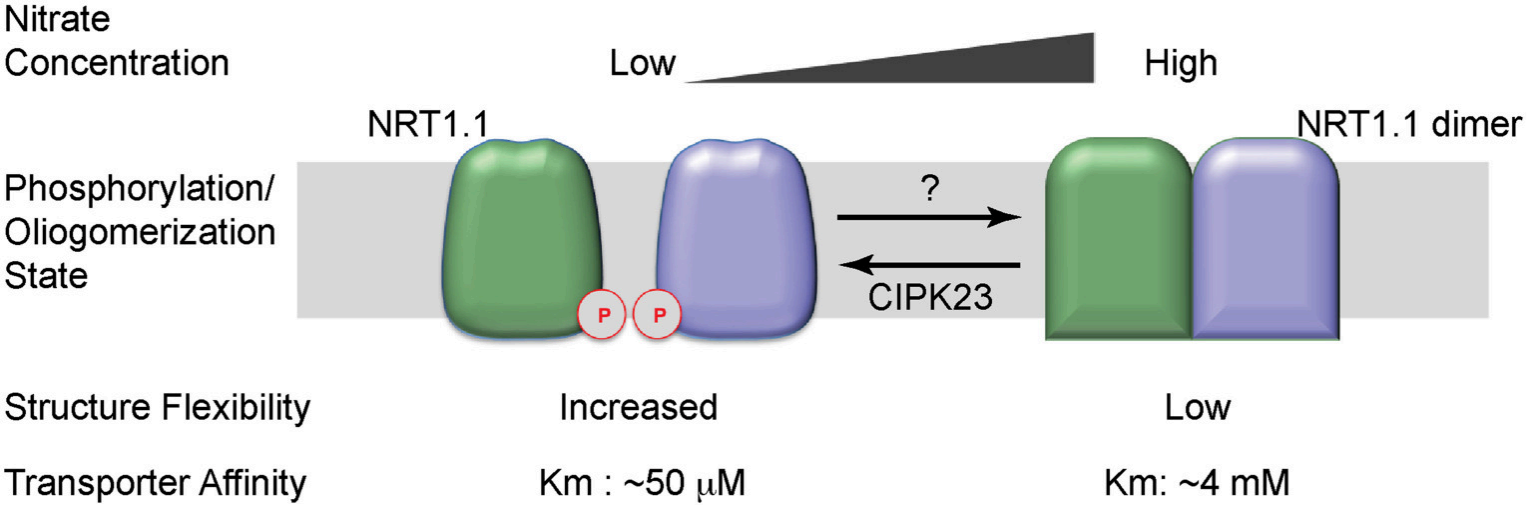
**Working model of the dual-affinity transporter, NRT1.1**. When nitrate concentration is low, NRT1.1 is phosphorylated (P) at the amino-acid residue Thr101. The phosphorylation modification disrupts the dimer configuration and increases the structural flexibility of NRT1.1, leading to a high affinity for nitrate. When nitrate is abundant, Thr101 is dephosphorylated. NRT1.1 works as a coupled dimer, which has decreased structural flexibility, rendering the transporter in a low-affinity state. The two NRT1.1 molecules are colored in pale green and light blue. The phosphorylation modification is indicated by “P” in a red cycle.

Taken together, phosphorylation on the Thr101 toggles NRT1.1 between the high- and low-affinity states by decoupling the dimer and increasing structure flexibility (Tsay, 2014; Figure 2). When nitrate is abundant, NRT1.1 is dephosphorylated. At this state, the transporter adopts a dimeric conformation, has lower structural flexibility, and functions as a low-affinity transporter. When the environmental nitrate concentration drops, NRT1.1 becomes phosphorylated on the Thr101 residue at the dimer interface, which decouples the NRT1.1 dimer. As a result, the phosphorylated transporter gains increased structural flexibility and uptake activity, and works as a high-affinity transporter.

### Substrate recognition and proton coupling in NRT1.1

A key question regarding to the working mechanism of a transporter is how substrate is recognized. High-resolution structures of bacterial nitrate transporter and nitrate/nitrite exchanger (NarU and NarK) from NNP family had been documented before the crystal structures of NRT1.1 were determined (Yan et al., 2013; Zheng et al., 2013). Nitrate in these two bacterial transporters is coordinated by two opposing conserved arginine residues through ionic interactions, which provides high-affinity contacts between the transporters and nitrate. Distinct from NarU and NarK, the overall substrate-binding pocket of NRT1.1 is mostly hydrophobic, with the exception of one histidine residue, His356 on TM7 (Figure 1B), which is shown to be critical in nitrate recognition. Despite the resolution limit, discernable electron density of nitrate is located in close vicinity to His356, mutation of which eliminated substrate uptake both at high and low nitrate concentrations. Notably, His356 is not conserved in transporters of the NRT1/PTR family, suggesting that other NRT1/PTR members may have different substrate recognition mechanism. The unique chargeable histidine residue at the substrate binding pocket provides a plausible explanation for the high-affinity activity acquired by NRT1.1. Sequence differences in the substrate binding sites may also explain why NRT/PTRs are able to recognize substrates as diverse as nitrate, peptides and plant hormones.

In addition to His356, an EXXER motif and a potential salt bridge between Lys164 and E476 (Figure 1B) are shown to be critical in proton coupling by uptake assays in liposomes and oocytes (Parker and Newstead, 2014; Sun et al., 2014). The importance of these residues is also validated by two independent assays, two-electrode voltage clamp (TEVC) and a novel fluorescent method, in which the activity of NRT1.1 was monitored through its tight correlation with FRET readout of fluorescent proteins linked to N- and C-terminus of the transporter (Ho and Frommer, 2014).

### MFS oligomerization

Besides NRT1.1, numerous MFS transporters including TetL (Safferling et al., 2003), GalP (Zheng et al., 2010), GLUT1 (Graybill et al., 2006), hRFC (Hou and Matherly, 2009), MCT8 (Fischer et al., 2015), and LacS (Veenhoff et al., 2001) have been shown to exist as homo-oligomers. Functional importance of the MFS oligomerization has been indicated in several studies. For example, the lactose transporter LacS from *Streptococcus Thermophiles* functions as cooperative dimers with two substrate translocation pathways, and co-reconstitution of functional and defective transporters disrupts proton-coupled lactose uptake, suggesting a dominant-negative effect (Veenhoff et al., 2001). The human monocarboxylate transporter 8 (MCT8), mutation of which underlies the cause of a severe X-linked psychomotor retardation (known as the Allan-Herndon-Dudley syndrome), forms functional homodimers *in vivo*. Native occurring pathogenic mutations from patients that abolish the transporter function also largely affect the formation of homodimers, suggesting the potential significance of the transporter oligomerization (Fischer et al., 2015).

In the case of NRT1.1, phosphorylation-controlled dimerization provides a novel aspect on how dynamic transporter oligomerization involves in functional modulation. The dimerization state of NRT1.1 is fine-tuned by the posttranslational modification. Upon phosphorylation and dephosphorylation of the Thr101 residue, the transporter oligomerization state is regulated, which in turn further changes the structure flexibility of NRT1.1 and switches the transporter between high- and low-affinity modes. Thus, structure-function studies of NRT1.1 not only establishes a structural framework for understanding the dual-affinity activity, but also reveals how posttranslational modification and protein oligomerization can synergistically expand the functional capacity of an MFS transporter.

### Perspective

In this review, we focused on the recent progress on the molecular basis of the dual-affinity activity of the key nitrate transporter, NRT1.1. Through structural and biochemical studies, the oligomerization state and the structural flexibility of the transporter are proposed to play a key role in the phosphorylation dependent transporter affinity switch. Yet many important questions remain to be answered to further our understanding on this important molecule. For example, what is the structure of the phosphorylated transporter like? Crystallographic analysis of the phosphorylated form will help us visualize the conformational difference between the high- and low-affinity states, and obtain a better picture of the working mechanism. Since the high-affinity property of NRT1.1 has been challenged (Glass and Kotur, 2013), it will be also valuable to systematically characterize the function of NRT1.1 at different oligomerization and phosphorylation states *in vitro*. By mutating and disrupting the dimer interface (Robertson et al., 2010), a phosphorylation-independent NRT1.1 monomer could be potentially obtained. Structural and functional analyses of such a mutant could help address the questions as to whether phosphorylation alters the properties of the transporter beyond controlling its oligomerization state and whether unphosphorylated NRT1.1 monomer is functional. Furthermore, how Thr101 becomes modified by the CIPK23 kinase if this residue is buried in a hydrophobic pocket at the dimer interface? It is proposed that the phosphorylation might occur when the transporter is at the occluded or outward conformation, yet this hypothesis awaits more evidence. Last but not least, do both protomer copies of the NRT1.1 dimer have to be phosphorylated before the affinity state of the transporter is changed? Designing a functional NRT1.1 concatemer with two individually manipulable copies of the transporter might help shed light on this and other questions.

Another interesting aspect of the NRT1.1 protein is its nitrate sensor function, which exemplifies the transceptor (transporter with sensor function) paradigm in *Arabidopsis* (Ho et al., 2009; Gojon et al., 2011). Transceptors have been found in many organisms from *E. coli* to mammals (Thevelein and Voordeckers, 2009), and almost all transceptors are important in nutrients transport and sensing (Donaton et al., 2003; Schwöppe et al., 2003; Hyde et al., 2007; Rebsamen et al., 2015; Wang et al., 2015). Recently, the mammalian amino acid transporter, SLC38A9 was identified as an amino acid sensor for lysosome-based activation of mTORC1, indicating the importance of transceptor in human disease (Abraham, 2015). A fundamental question underlying the working mechanism of a transceptor is how substrate translocation and sensing is coupled? In the case of NRT1.1, its transceptor function also exhibits a biphasic manner switched by Thr101 phosphorylation, as the phosphorylation and non-phosphorylated forms of NRT1.1 have distinct signaling properties (Bouguyon et al., 2015). It will be interesting to know whether NRT1.1 dimerization is also involved in the sensor affinity switch. An equally important question is how nitrate signal is transduced through protein-protein interaction. In the crystal structure of NRT1.1, the well-ordered N-terminal loop at the dimer interface on the intracellular side presents a conserved docking interface that may have a putative role in nitrate signaling by recruiting downstream signal molecules such as kinases and phosphatases. Further genetic and biochemical studies will be needed to support this hypothesis.

Last but not least, NRT1.1 facilitates nitrate-regulated auxin transport. It has been reported that NRT1.1 could use both nitrate and auxin as its substrates, and enable soil nitrate availability to regulate the lateral root development (Krouk et al., 2010b). This reveals a surprising mechanism by which plants adjust their root architecture for soil exploitation, and raises another interesting question as to how NRT1.1 recognizes two structurally different substrates. Further studies will be required to fully uncover the role of NRT1.1 in nitrate transport and signaling, which enable plants to adapt to the fluctuated nitrate environment. Ultimately, these studies will potentially facilitate the development of new technologies for increasing crop yields as well as reducing nitrogen pollution in modern agriculture.

## Author contributions

Both authors contributed to the drafting and revising the work.

## Funding

This work is supported by the Howard Hughes Medical Institute and the National Science Foundation (NSF MCB-1157561).

### Conflict of interest statement

The authors declare that the research was conducted in the absence of any commercial or financial relationships that could be construed as a potential conflict of interest.
